# Self-Broadening of Carbon Monoxide in the 2 *v* and 3 *v* Bands *

**DOI:** 10.6028/jres.067A.022

**Published:** 1963-06-01

**Authors:** Earle K. Plyler, Robert J. Thibault

## Abstract

The self-broadening of carbon monoxide has been measured for the 2 *v* and 3 *v* bands with pressures up to 3.5 atmospheres. A grating spectrometer of high resolving power was used for the measurements and the correction for finite slits was small. The corrections varied from 3 to 20 percent for the different conditions of measurement. The half-widths per atmosphere, *γ°*, decreased from 0.089 cm^−1^ for |*m*| = 1 to 0.053 cm^−1^ for |*m*| = 21. The half-widths are compared with those obtained by other investigators and it is shown that the results reported in this work fall in between the self-broadening values previously obtained.

## 1. Introduction

The broadening of the rotational lines of carbon monoxide by foreign gases has been investigated for a number of gases such as CO_2_[1].^1^ The observed half-width, *γ*, is the sum of the self-broadening of CO and the foreign gas broadening. This may be expressed as follows:

$$\gamma =\gamma^{0}{\;}_{\text{CO}}P_{\text{CO}}+\gamma^{0}{\;}_{F}P_{F}$$ (1)

where *γ*^0^_CO_ is the half-width per atm for CO selfbroadening and *γ°_F_* is the half-width per atm of the foreign gas broadening; *P*_CO_ and *P_F_* are the partial pressures in atmospheres of CO and of the foreign gas, respectively.

If a long path cell of 4 m or greater is used, the partial pressure of CO which produces optimum line blackening is a few mm (Hg) or less and the term, *γ*°_CO_*P*_CO_, of eq (1) approaches zero. Whenever a long path cell is available, more accurate results for *γ*^0^ can be obtained. If a cell with an optical path of 10 cm is used, the pressure of the CO may have to be of the same order of magnitude as the pressure of the broadening gas to obtain suitable measurements. In such cases the self-broadening of the carbon monoxide must be known before *γ*^0^ for the foreign gas can be determined. If the foreign gas broadening is smaller than the self-broadening of CO, appreciable errors may be introduced by using inaccurate values for the CO self-broadening.

The self-broadening of CO has been measured in three different laboratories and the values differ somewhat. This work was undertaken to determine a precise set of values for the self-broadening of CO.

## 2. Experimental Procedures

In order to secure the best possible results, a high- resolution grating spectrometer was used double- passed in this work. A 10,000 1/in. commercial replica grating was used in the first order for the 2–0 band and in the second order for the 3–0 CO band. An optimum balance between slit-width, response time and noise level was obtained with a spectral slit of 0.08 cm^−1^. A response of 98 percent of the total deflection was obtained in 1 sec in the 2–0 and 3–0 bands. The grating was driven at 18 sec of angle per minute of time which was about 0.06 cm^−1^/min in the 2–0 band. Further information regarding the spectrometer and its operation can be obtained from a previous publication [2].

Several preliminary tests were made before the broadening was measured. These included slit width measurements, stray energy tests, transmission tests, and recorder linearity checks.

Many different path and pressure combinations were employed to insure the reliability of our results. These are listed in table 1. The higher pressures involved were measured with a Bourdon tube type gage with a stated accuracy to within percent at 100 lb/in^2^.

The correction of the observed half-widths for the finite slit width of the spectrometer was made using the correction factors proposed by Izatt [3].

## 3. Results

The half-width of a spectral line may be defined as half its physical width, measured at the frequency corresponding to half its maximum extinction, expressed in reciprocal centimeters per atmosphere. The method of measurement used in our work is essentially the same as that applied previously in this laboratory (see ref. 1). It is a very simple method consisting of measuring the line widths at 40, 50, and 60 percent of the extinction coefficient at maximum absorption. The three values obtained are reduced to the half-width for 50 percent of the maximum coefficient by dividing by a constant which is dependent on the coefficient of extinction and line shape. These factors are 1.22 for 40 percent, 1.0 for 50 percent and 0.817 for 60 percent, assuming Lorentzian line shape. Since it is a direct method, and the slit corrections are relatively small (<20 percent in all cases and <7 percent in most), our results should certainly be considered reliable to within ± 5 percent.

**Figure 1:**
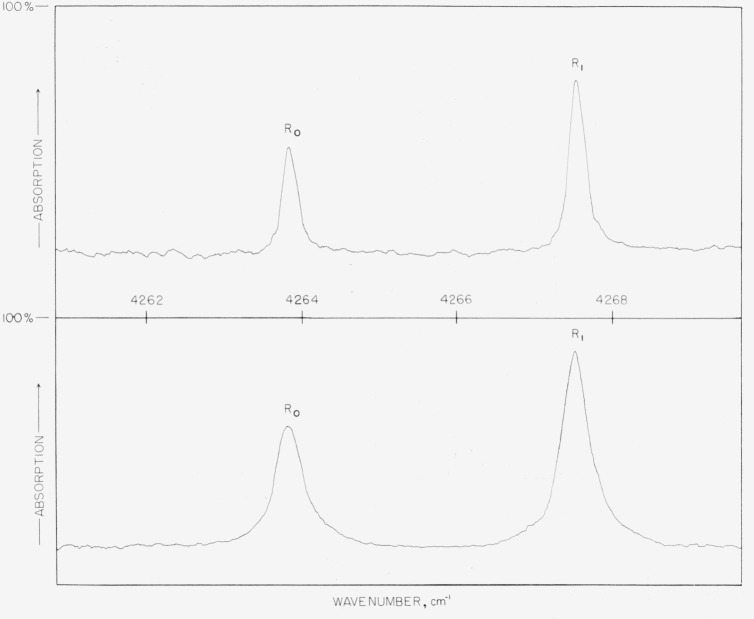
The upper curve shows the self-broadening *CO* lines *R*_0_ and *R*_1_ of the 2 v band under 1 atm pressure. The lower curve shows the same lines with 2.1 atm pressure. The absorption path length in both cases was 9.0 cm.

**Figure 2:**
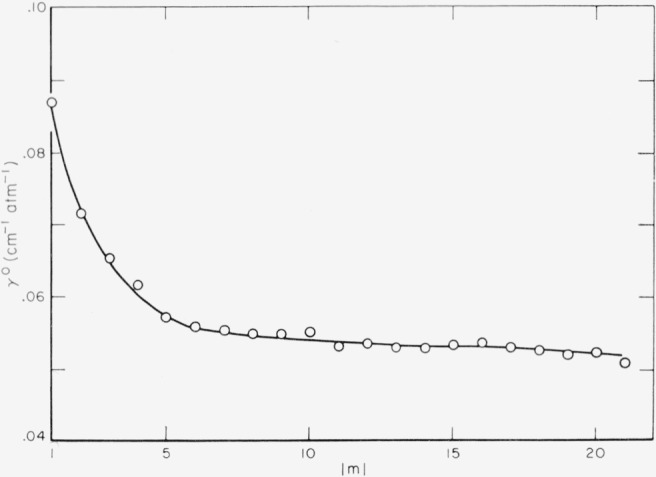
The variation of the half-widths, γ°, for carbon monoxide as a function of the rotational number |*m*|.

An example of the data obtained, showing the effect of pressure change on self-broadened CO lines, is given in figure 1. The half-widths measured from these data are presented in the form of a table and a graph. Table 2 lists the average values obtained for both the (2–0) and the (3–0) CO bands separately, while figure 2 represents the weighted average of all values. The self-broadening, *γ°*, of corresponding lines (same |*m*|) of the *P* and the *R* branches agreed within experimental error and are averaged in the final results.

**Figure 3:**
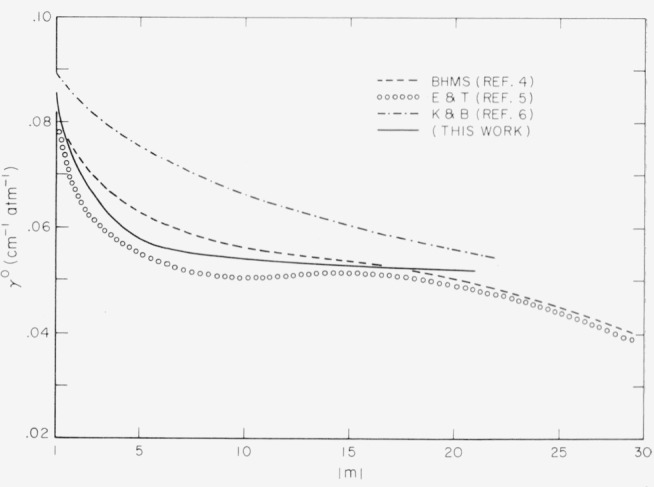
A comparison of the half-widths, γ°, for carbon monoxide as obtained by different observers.

The self-broadening of carbon monoxide has previously been measured in three laboratories. Table 3 gives a brief summation of the conditions of measurement. The results obtained by each group are plotted in figure 3, along with the measurements reported in this paper.

## 4. Discussion

The results obtained in this study fall between those of Benedict et al. [4] and those of Eaton and Thompson [5]. The greatest variation of their values from our results occurs at |*m*|=7 where the disagreement is about 8 percent. The average of the two sets of values from |*m*| = 1 to |*m*| = 16 fall very close to our results. The half-widths reported by Kostkowski and Bass are somewhat larger than those reported by other workers. Since their work was extended to higher pressures, the inherent overlapping in the wings of lines may have influenced their results.

It should be noted that although data were obtained for lines of *J*>21, these are not considered as reliable as the low *J* values, due to their low intensity, and have not been included. No quantitative conclusion can be drawn from the present work as to decrease in half-widths with higher *J* values; however, the variation of *γ°* with |*m*| must be related to the directional properties of the intermolecular forces. In the case of self-broadening in CO, it is believed that both the quadrupole-quadrupole and anisotropic dispersion forces are of primary importance. The rapid decrease in *γ°* with |*m*|, and the magnitude of *γ°* have been approximately accounted for by a recent calculation based on these forces [7].

Quantitative measurements of the intensities of the lines of 2 *v* (4260 cm^−1^) and 3 *v* (6350 cm^−1^) bands of CO were not made. However, it was observed that the intensities of the *P* and *R* branches of the 2 *v* compared with those of the 3 *v* were approximately equal when a pressure of 3 atm and 400 cm path length were used for the measurement of 3 *v* and 1.5 atm pressure and 4 cm path length were used for 2 *v*. This indicates that the intensity of 2 *v* compared to 3 *v* is roughly 200 to 1.

## Figures and Tables

**Table 1:** Pathlengths and pressures used in measurement of CO self-broadening

2–0 Band		3–0 Band	
Pathlength	Pressure	Pathlength	Pressure
			
*cm*	*atm*	*cm*	*atm*
4	1.65	400	2.00
4	1.83	400	2.29
4	3.45	400	3.00
9	1.00		
9	2.00		
9	2.17		
9	2.34		
9	3.00		
100	2.00		

**Table 2:** Half-width, γ°, for self-broadening of carbon monoxide for the 2 v and 3 v bands

|*m*|	γ° (cm^−1^ atm^−1^)		Weighted average	No. of measurements
	2–0	3–0		
				
1	0.0870	………	0.0870	9
2	.0712	0.0732	.0716	10
3	.0651	.0665	.0654	13
4	.0605	.0643	.0616	7
5	.0567	.0582	.0572	6
6	.0553	.0569	.0560	7
7	.0546	.0568	.0555	6
8	.0543	.0564	.0551	8
9	.0548	.0556	.0551	6
10	.0553	.0552	.0552	8
11	.0533	………	.0533	4
12	.0538	.0534	.0536	7
13	.0538	.0523	.0532	5
14	.0529	.0528	.0529	4
15	.0534	………	………	4
16	.0535	………	………	5
17	.0531	………	………	5
18	.0528	………	………	5
19	.0522	………	………	4
20	.0523	………	………	1
21	.0512	………	………	2

**Table 3:** The conditions of measurement of the self-broadening of *CO*

Investigator	Lines reported	Pathlength	Pressure Hg	Slit width	CO band
					
		*cm*	*cm*	*cm*^−1^	
Eaton & Thompson	|*m*|=1 to 28	0.6 to 20	1 to 30	0.5	(1–0)
Benedict et al	|*m*|=1 to 32	0.03 to 890	0.3 to 228	0.2	(1–0) and (2–0)
Kostkowski & Bass	|*m*|=1 to 22	1 to 100	380 to 760	0. 25	(2–0)
This work	|*m*|=1 to 21	4 to 400	76 to 262	0.08	(2–0) and (3–0)

## Footnotes

1 Figures in brackets indicate the literature references at the end of this paper.
